# Supplementary material for: A framework to determine active neurons and networks within the mouse brain reveals how brain activity changes over the course of the day
Source: PLoS Biol. 2025 Nov 13;23(11):e3003472. doi: 10.1371/journal.pbio.3003472 (PMC12633928; doi:10.1371/journal.pbio.3003472)
Supplement: Attachment — Submitted filename: active_connectivity_review_response.pdf [file pbio.3003472.s001.pdf]

# Response to Reviews for Manuscript: A framework to determine active neurons and networks within the mouse brain

Guanhua Sun[1], Tomoyuki Mano[4, 1], Shoi Shi[4], Alvin Li[1], Koji L Ode[4], Alex Rosi-Andersen[3], Erica Pedron[3], Steven A Brown[†3], Hiroki R Ueda[4, 5, 6], Konstantinos Kompotis[*3, 1], and Daniel Forger[*1, 2]

[1]Department of Mathematics, University of Michigan, Ann Arbor, United States
[2]Gilbert S. Omenn Department of Computational Medicine and Bioinformatics, University of Michigan, Ann Arbor, United States
[3]Chronobiology and Sleep Research Group, Institute of Pharmacology and Toxicology, University of Zurich, Zurich, Switzerland
[4]Department of Systems Pharmacology, Graduate School of Medicine, the University of Tokyo, Tokyo, Japan
[5]Laboratory of Synthetic Biology, RIKEN Biosystems Dynamics Research, Osaka, Japan
[6]Department of Systems Biology, Institute of Life Science, Kurume University, Fukuoka, Japan

† In memoriam
∗ To whom correspondence should be addressed

# Letter to Reviewers and Editors

Dear Reviewers and Editors at PLOS Biology:

Thank you for reviewing our manuscript! We sincerely appreciate the time and effort you have dedicated to this process.

After more than a year of effort, we are pleased to present our revised manuscript titled **"A framework to determine active neurons and networks within the mouse brain"**. Before going to the point-to-point response, we would like to highlight the improvements we have made to the manuscript based on your comments.

For methodology, we have made the following major improvements:

1. We have increased the number of experiments by 4-5 times. Compared to the two time windows (ZT0-4 and ZT12-16) we chose in the original manuscript, new experiments now cover four different time windows (ZT0-4, ZT8-12, ZT12-16 and ZT20-24), including both the beginning and the end of the light or the dark period. This increase in time windows diversifies our observations of active neurons and networks.
2. We have more than doubled the sample size at each time point (from N = 2 to N = 4 or 5). This increase in sample size provides more statistical support for our findings.
3. We have deepened our analysis of the distribution of the active neurons, which was missing in our original manuscript.
4. We introduced a new method to match the identified active neurons to a more recent spatial transcriptomic dataset in the Allen Brain Atlas [1]. Compared to the cell atlas we originally used [2], the new method has significantly increased the resolution and accuracy of identifying different neuron types. As more and more spatial transcriptomic datasets are becoming available, we believe this addition greatly increases the usability of our framework.
5. We have refined our definition of "active connectivity." Compared to the original manuscript, we no longer build a neuron-to-neuron connectome between active neurons, but build a mesoscopic, regional active connectivity matrix for each brain. This improvement makes it possible to use established tools from network science to systematically analyze the derived active connectivity across different time windows. A new appendix has been added to explain the mathematical basis of the construction and analysis of the active connectivity.

The changes in our methodology have brought many interesting new discoveries. Here is a rundown of our new findings presented in the revised manuscript:

1. Among the major brain regions, we identified that the active neurons in the cortex have the most significant fluctuation across the four time windows. This is shown in Figure 2.
2. Within the cortex, we observe region-specific variations of active neurons, especially in the visual cortex (VIS), posterior parietal association areas (PTLp), and the prelimbic

areas(PL). These differences are shown in Figure 3.

3. Surprisingly, we also observed a robust layer-specific difference in the distribution of active cortical neurons across the four time points. Layer 5 neurons are relatively much more active at the end of the dark period (ZT20-24), compared to other time points, especially ZT12-16. These findings are shown in Figure 4.

4. By using the new method and the spatial transcriptomic dataset aforementioned, we identify the molecular information of the active neurons (Figures 5 and S5). We then find that the excitatory/inhibitory ratio of active neurons in the brain changes significantly across the four time points, especially in the thalamus, but not in the cortex (Figure 6).

5. Active connectivity is built for each brain (Figure 7). The modularity of each active connectivity reveals structural differences across the four time points (Figure 8). Furthermore, by calculating the centrality of different regions within the active connectivity, we find how hubs shift from subcortical structures such as the posterior hypothalamic nucleus during ZT12-16 to cortical regions that belong to the default mode network in ZT20-24 (Figures 9 and 10).

We hope these improvements and new findings have greatly improved the validity of our framework and the quality of the manuscript. As we have written at the end of the revised manuscript, as more multimodal neuronal data are becoming available at an increasing speed, we believe the framework presented in the revised manuscript is a great demonstration of how to combine these data with experiments to advance the exploration and analysis of the mouse brain [3][4].

The manuscript has been carefully rewritten, and we hope your comments are well-addressed. We look forward to hearing back from you on our revised manuscript.

Sincerely,

Authors

# Point-to-point response to reviews

## 1 Reviewer 1

The manuscript by Sun et al. "A Framework to Determine Active Connectivity within the Mouse Brain" described a new method to construct state-dependent network connectivity by integrating neuron activity and structural connectivity in the brain. The inference of network connectivity in the brain is an important topic in neuroscience. The circadian rhythm/sleep is also an excellent venue to apply the network construction methodology. However, the novelty in the method and the insights obtained in the application in the circadian/sleep neural network should be more clearly described. The followings are my comments:

**Point 1.1**

The definition of "active connectivity" is unclear. In principle, both excitations and inhibitions are present in the neural network. Therefore, the active and inactive neurons in all time points should be considered together rather than be treated separately as it is now. Based on the method described in Page 6-7 and those in methods section, it appears that the method is simply based on the distributions of active/inactive neurons in the regions involved in structural connections.

**Response 1.1**

Thank you very much for your comments. We acknowledge that the original definition of active connectivity was unclear. In the original manuscript, we were aiming to build a connectome that consists only of active neurons, which can be directly simulated to potentially explain different behaviors or network dynamics at different times of the day. We then did many simulations with both active and inactive neurons, but these results are too complicated to interpret with our data at this stage.

Inspired by your and the editor's comments, we realized the most important thing can be shown from our data was probably how active neurons formed networks that are related to known networks in the brain, such as the default mode network, as you have pointed out later. This prompts us to rethink the following questions: knowing the number of active neurons in each region and the underlying mesoscopic structural connectivity, how can we measure the structural change of the networks formed by these active neurons? How do the roles of different regions shift in this network at different times of the day?

To answer these questions, we refined our definition of active connectivity and used established tools from network theory to study these networks. We came up with a metric that measures the communication between active neurons in regions A and B, based on the following three quantities:

1. Regions A and B are structurally connected with a connection density $\rho_{AB}$,
2. Region A contains a sufficient number of active neurons ($N_A$) to send signals,
3. Region B contains active neurons ($N_B$) receiving the signal and sending it to a different region.

We then assume the "active connection strength" between region A and region B is the product of these quantities: $W_{AB} = N_A \times \rho_{AB} \times N_B$. This definition will result in a different connectivity matrix for each brain, which can be analyzed like other studies have done for the structural connectivity [5] [6]. We have done so in this manuscript and have revealed many insights about how the network formed by active neurons changes across different times of the day, which are shown in our new Figures 8 - 10. Please also see the appendix for more details about the construction and analysis of the new active connectivity.

During our revision, we did consider using the spatial transcriptomic dataset (Zhuang-ABA1) [1], which is also used in this manuscript, as a reference for all neurons in the brain to include the inactive neurons into consideration. This means that we would treat the neurons in this dataset as the background of all the neurons in the brain. However, 2.5 million neurons are still only a small portion of the total population, which is about 80 million [7]. Certainly, the study we present here will be more inclusive if a different experimental protocol is adopted to identify both the active and inactive neurons, which we have addressed in the discussion:

> Based on the number of neurons we identified, our experiments are capturing only about the most active 1% of the total 80 million neurons contained in the mouse brain [7], ignoring the vast majority of inactive neurons. Nevertheless, one of the main strengths of this framework is the modular manner in which it was built, such that it can integrate whole-brain data from both endogenous and exogenous fluorescence, as well as different whole-brain clearing techniques, and match them with novel spatial transcriptomic datasets of interest. To include specific neuronal populations, alternative experimental designs involving labeling of active or inactive populations via staining of expressed proteins of interest can be of complementary value to the analysis conducted here. Moreover, although we chose to focus on four time windows around the 24-hour day with distinct circadian, light and sleep-wake driven behaviors, this framework can also be extended to study mouse brain connectivity under many other conditions, including neurodegenerative states, behavioral interventions, sleep deprivation, or even in combination with automated systems, such as the IntelliCage system [8].

We hope these have addressed your questions.

**Point 1.2**

The whole-brain comparison of active/inactive neurons between ZT0-4 and ZT12-16 is more straightforward. How the method described here is different from the connections between the regions more active at ZT0 and the regions more active at ZT12?

**Response 1.2**

Thank you for raising this insightful question. First, we have strengthened our analysis of the active neurons at different time windows of the day, which have increased from 2 to 4 windows.

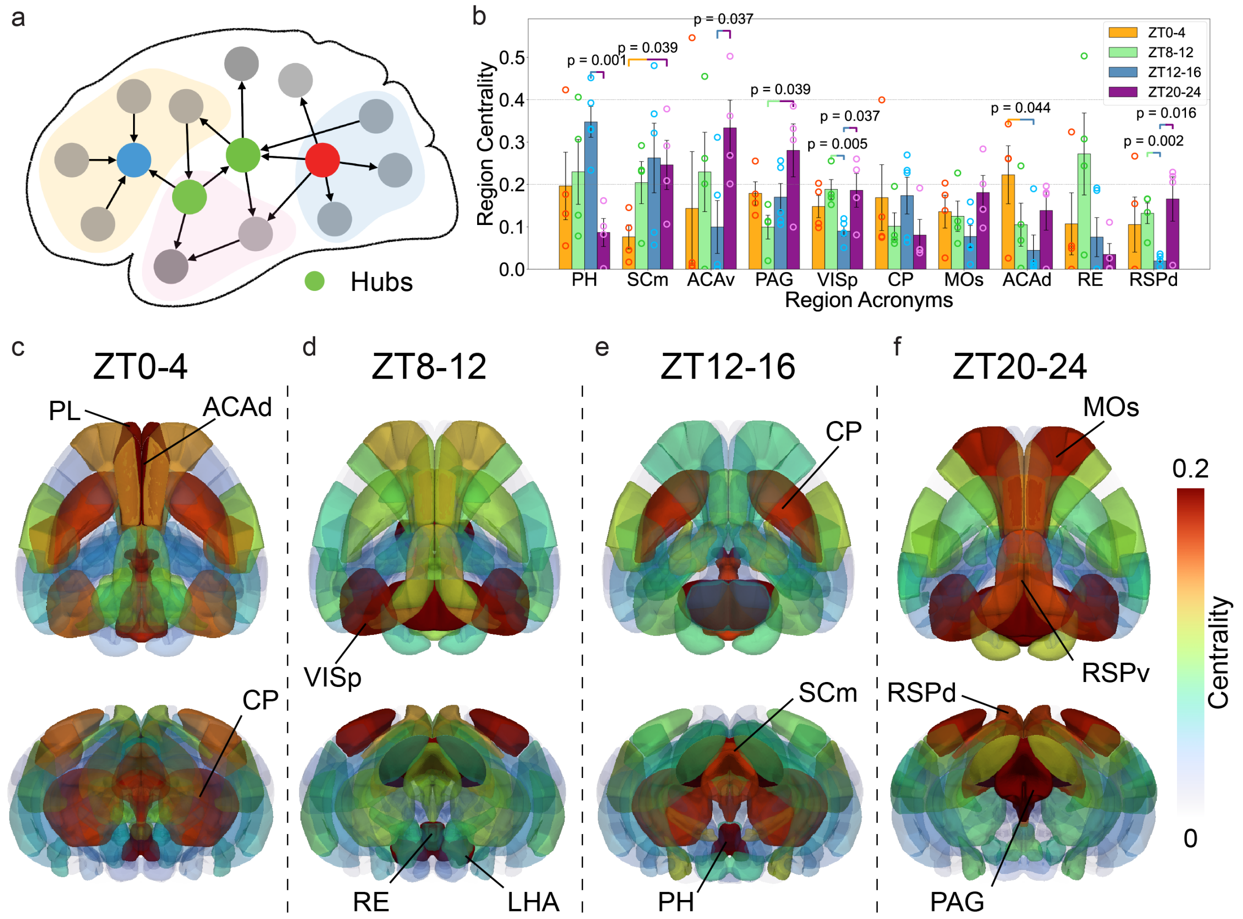


Figure 9: **Active hubs in the brain at different time windows.** a) We identify the hubs within the active connectivity by calculating the betweenness centrality of different regions (Methods). b) The top 10 regions with the highest centrality on average across the four time windows c-f) Visualization of different brain regions, which are colored based on their centrality at different time windows (left to right: ZT0-4, ZT8-12, ZT12-16, ZT20-24) and from different views(top: horizontal, bottom: coronal).

Regarding how the network comparison is different from the direct analysis of the active neuron in different regions, we would like to illustrate by pointing to **Figure 10** (attached below) in our revised manuscript about the analysis of the posterior hypothalamic nucleus. From the figure, you can see that although the number of active neurons in the posterior hypothalamic nucleus stays relatively the same across the four time points, the regional centrality has changed a lot. This is also addressed in the results section:

> It may seem intuitive that an increase in the number of active neurons would correspond to a simultaneous increase in both the strength and centrality of a brain region. However, this is not necessarily the case. For instance, the posterior hypothalamic nucleus exhibits no significant change in the number

of active neurons across the four time windows (Fig. 10e). Yet, its regional centrality shifts significantly between ZT12–16 and ZT16–20 (Fig. 10f). These findings collectively emphasize the utility of active connectivity, beyond analyzing regions alone, in capturing temporal shifts in brain network organization and identifying key regions whose activities fluctuates through different times of the day.

To summarize, the network measures we implemented in the revised manuscript, including modularity and betweenness centrality, are both nonlinearly dependent on the number of active neurons in the source and the target region. Therefore, our analysis of the constructed active connectivity can bring in new insights that direct analysis of the regions can not produce. We would also refer to our Fig. 9 (attached in the previous page), which shows how centrality analyses reveal the hubs switch between different time windows. We hope these have clarified your question.

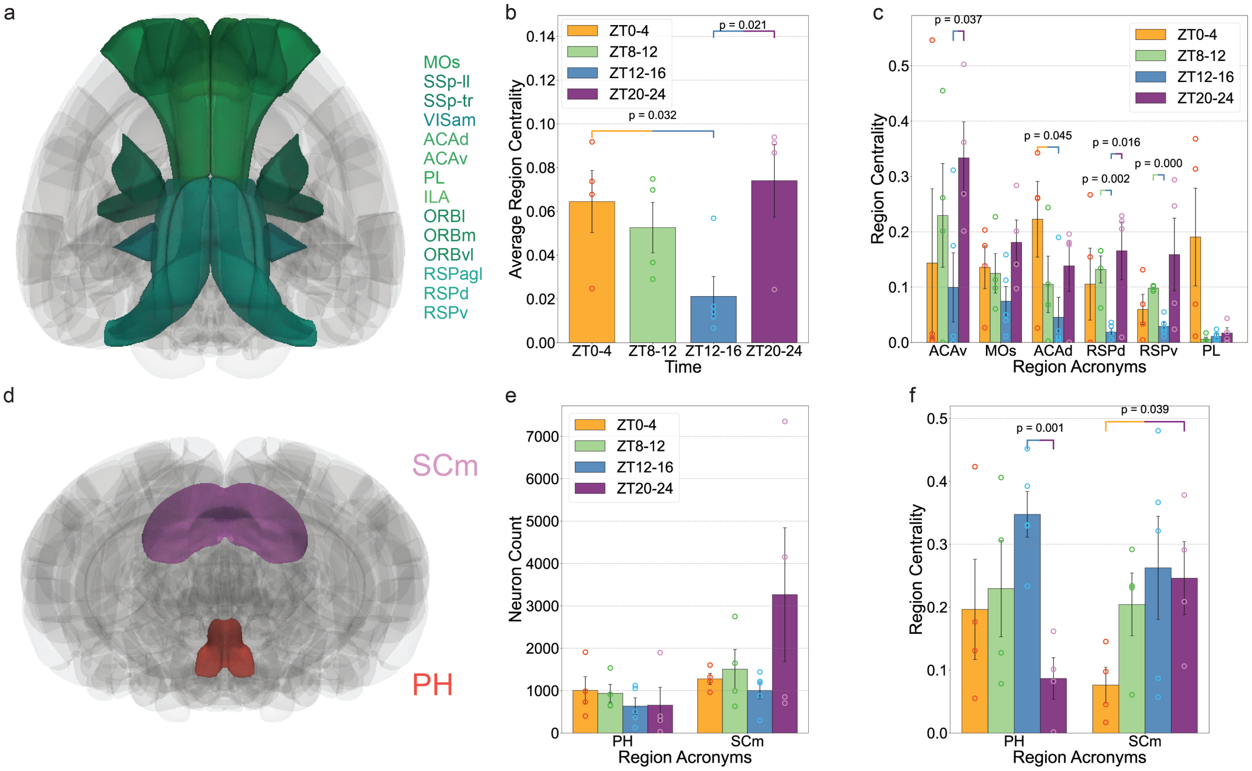


Figure 10: **Centrality of the default mode network (DMN), motor-related superior colliculus (SCm) and posterior hypothalamic nucleus (PH).** a) Positions of different DMN regions in the whole brain (Horizontal view). b) The average centrality of DMN regions at different time windows. c) Centrality of the top 6 DMN regions at different time windows. d) Positions of SCm and PH in the whole brain (Coronal view). e) Number of active neurons in PH and SCm at different time windows. f) Centrality of SCm and PH at different time windows.

Point 1.3

The numbers of samples were small (i.e., N=2 ZT0, and N=2 ZT12) which has limited the statistical power to test the difference of active or inactive neurons between the two time points.

Response 1.3

Thank you for raising this important concern. We acknowledge that the sample size in the original manuscript limited the statistical power of our analyses. In the revised manuscript, we have addressed this by increasing the number of subjects per time point from two to four or five (N = 4–5), which yields adequate statistical power for our approaches, also supported by previous studies [9]. Furthermore, all findings presented in the revised version are now supported by appropriate statistical tests.

Point 1.4

The authors assigned the active neurons to be either excitatory or inhibitory based on the E/I distribution in Ref. 21. This would be technically difficult in the regions with intermixed excitatory or inhibitory neurons, such as Layer 2/3 of cortex where both pyramidal neurons and interneurons exist. Furthermore, how the assignment of excitatory or inhibitory neurons played any role in the subsequent network reconstruction? This was not described in the paper.

Response 1.4

Thank you for this insightful comment. We agree with your assessment that our original method, combined with the data from [2], limits the resolution needed to distinguish between different neuron types. Addressing this limitation has become a central focus—and we believe, an innovation—of our revised manuscript. Specifically, we now leverage the spatial transcriptomics dataset from the Allen Brain Atlas (Zhuang-ABA1), which provides molecular profiles of cells in the mouse brain on single-cell resolution, in contrast to the regional resolution offered by Eros et al. To integrate this data into our framework, we have developed a new computational approach to map neurons in our dataset to those in Zhuang-ABA1, which is demonstrated in **Figure 5** (attached below).

We acknowledge, however, that this method still has limitations, particularly due to the complex spatial organization of diverse neuronal types in the mouse brain, especially in the isocortex, as you have pointed out. These challenges are discussed in detail in the revised Discussion section.

> The regional analysis of active neurons was followed by alignment to the spatial transcriptomic data by finding the closest neighbor in the dataset (Zhuang-ABA1). Although the spatial distribution between neurons in our dataset and the matched neurons is relatively close, the method can still produce discrepancies, in particular, for those regions where different types of neurons are tightly mixed, such as the cortex. Our method will work especially well for brain regions where different types of neurons are more spatially segregated,

such as the thalamus or hypothalamus. In the future, we would expect more spatial transcriptomic data with higher resolution that can be seamlessly used in the framework presented in this work to further increase the accuracy of identifying cell properties [4].

For the second question, the neurotransmitter information was used in the cell-type-specific analysis of the active neurons, but was not used in constructing the active connectivity. This is also related to our response to your later point 1.6, which discusses the potential use of cell-type-specific structural connectivity information. We hope these answers have addressed your questions.

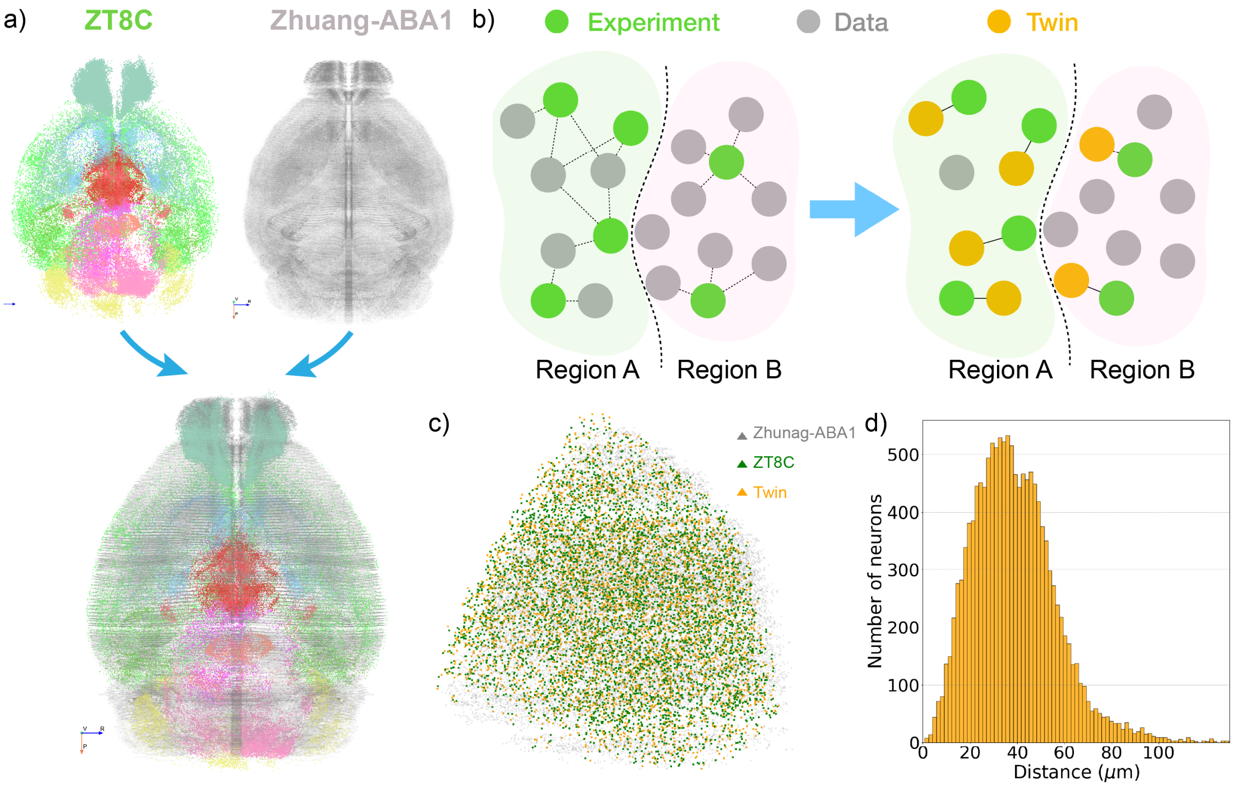


Figure 5: **Matching active neurons to the spatial transcriptomic dataset.** a) Spatial distribution of the neurons in Zhuang-ABA1(grey), our ZT8C brain(colored) and merged together in the same coordinate framework. b) Schematics of finding the twin neuron in the spatial transcriptomic dataset(Zhuang-ABA1) for each active neuron in our data based on spatial proximity. c) A demonstration of the neuron matching process with the right primary visual cortex of the ZT8C brain. Green points are our data, grey points are Zhuang-ABA1 and yellow points are the twin neurons after matching. d) The distribution of distances between the twin neurons in Zhunag-ABA1 and the neuron in our dataset for the ZT8C brain.

### Point 1.5

The description of the results of whole-brain comparison of active/inactive neurons is intriguing. In Page 12, it was stated "Neurons in VISp are upregulated in the morning because the lights are on and downregulated after the lights are off." In Page 15, it was then stated: "The third most activated structure in our dark period dataset was the visual cortex, which is necessary for spatial navigation." This appeared contradictory.

### Response 1.5

We appreciate you pointing this out. Indeed, our original description of this finding lacked clarity, and we thank you for highlighting the issue. As noted in your earlier comment (1.3), this seemingly contradictory result was likely influenced by the small sample size (N = 2) in the original manuscript. In the revised version, this limitation has been substantially addressed through an increase in both the number of time windows and the number of subjects per time point.

In the updated analysis, the visual cortex continues to emerge as a region exhibiting significant changes across the four time windows, as shown in **Figure 3** (attached on the next page). We quote our description of this finding here:

> Notably, the visual cortex showed a significant decrease in activity during ZT12-16 compared to ZT8-12, indicating a strong light-off response (Fig. 3c). In contrast, the difference between ZT20-24 and ZT0-4 was less pronounced, suggesting a weaker light-on response, a phenomenon previously reported in the literature [10]. On average, visual areas were more active during the light period (ZT0–4 and ZT8–12) than the dark period (ZT12–16 and ZT20–24).

We hope the revised results and figures provide a clearer and more consistent picture in response to your concerns.

### Point 1.6

The author mentioned in "limitation of our methodology" ("Secondly, while the statistical model of the physical brain connectivity represents a heroic effort, it does not represent a complete mapping of the neurons within the mouse brain."). As the authors pointed out, the voxelized connectome model from Knox was based on a rather coarse connectivity matrix, which would limit the spatial resolution of voxels used for this study. Given the more recent Allen Mouse Connectivity dataset with new cell-type-specific antegrade labeling results (Harris et al., 2019, Nature), the voxelized connectome model from Knox et al. may need to be updated.

### Response 1.6

Thank you for pointing this out. We have carefully considered how the newer dataset might refine our findings and have incorporated it into our analysis where feasible. We fully agree that the cell-type-specific connectivity data from Harris et al. [11] can be integrated with our framework and will offer valuable insights that could enhance our discoveries.

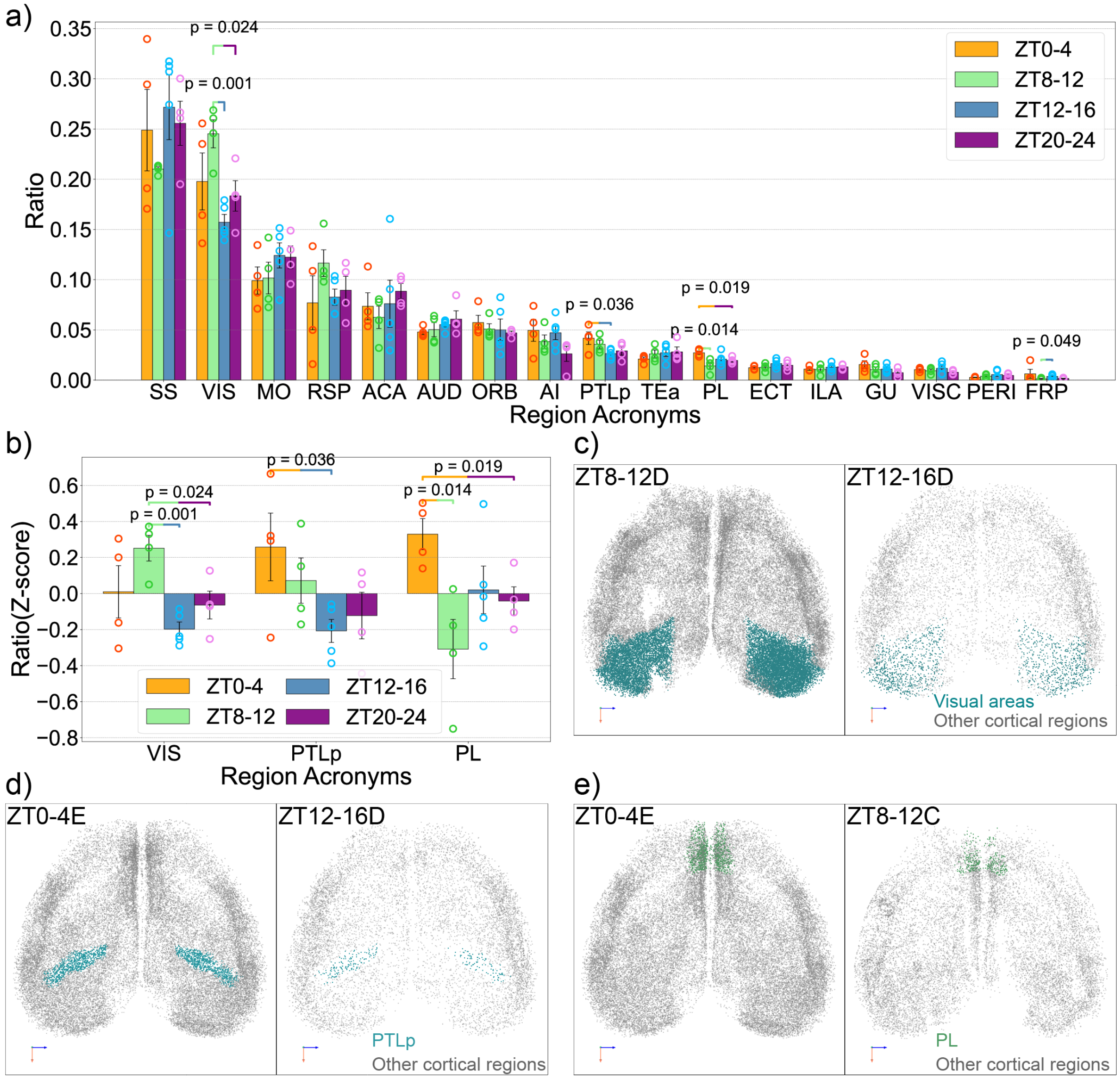


Figure 3: **Region-specific variation of active neuron distribution in the cortex.** a) Ratios of major cortical regions over the whole cortical population. b) The three regions that show significant differences in their ratios over the total cortical population across different times (VIS: Visual areas, PTLp: Posterior parietal association area, PL: Prelimbic areas). Here, the ratios are Z-scored based on the average of each region to show the differences between different times. c) Visualizations of active neurons in the visual regions for ZT8-12D and ZT12-16D brain. d) Visualizations of active neurons in the visual regions for ZT0-4E and ZT12-16D brain. e) Visualizations of active neurons in the visual regions for ZT0-4E and ZT8-12C brain.

However, this dataset is restricted to the thalamocortical circuit and does not cover other brain regions included in our study. As such, while it provides useful information, its limited scope prevents full integration into our current analysis. We view this as an important direction for future work, as emphasized in the revised Discussion section.

> To construct the active connectivity, we utilized one of the early versions of the whole-brain mesoscopic connectivity dataset [12]. Although a more recent update to this dataset is available [11], providing layer-specific and cell-type-specific projection information, it is limited to the thalamocortical network and therefore was not used for the whole-brain analyses conducted in this study. We also did not incorporate the voxelized extension of the connectivity data, which could significantly improve the spatial resolution of the results presented in this work. Integrating these resources represents an important direction for future work. Also, due to experimental procedures, we excluded the olfactory areas, cerebellum and medulla from the analysis. Missing these regions can cause discrepancies in analyzing the brain networks, and thus, including these regions in future studies will be valuable.

### Point 1.7

What is the relationship between the spatial distribution of active/inactive neurons and structural connections? Do the neurons in two structurally connected regions tend to be active/inactive together or not?

### Response 1.7

Thank you for raising this great question. First, we would like to say this is a highly non-trivial question and is frequently addressed in functional connectivity studies, where correlation of functional data with high temporal resolution is calculated to study the functional relationship between different brain regions under different behaviors and conditions.

As pointed out by the editors from PLOS Biology, it is difficult for us to collect "sufficient number of time points available for a covariation analysis even in the expanded dataset," which we think is true. Although we have expanded the number of time windows from 2 to 4, the number of observations at each time point (N = 4 or 5) is still too few to draw similar analyses that are usually conducted in functional connectivity studies.

However, with our data and computational method, together with the connectivity data provided by the Allen Brain Atlas, the question we can address is, after knowing which neurons have been active in the past four hours in different brain regions, what does the average network dynamics between active neurons look like? These thoughts, along with your comments, have made us redefine the active connectivity, which we have responded to your earlier point 1.1 . We hope these have addressed your questions and we are happy to conduct some covariation analysis with our data if you think it would be helpful.

**Point 1.8**

How are the changes of active/inactive neurons in different brain networks, such as Default Mode Network, Salience Network, and so on?

**Response 1.8**

Thank you for asking this important question, which naturally arises in our analysis of active connectivity. After we construct the active connectivity for each brain and analyze the centrality of each region, we found that many regions in the default mode network plays a much more central role in the whole-brain network at the end of the dark period (ZT20-24), compared to the earlier points, especially the beginning of the dark period (ZT12-16). As we have pointed out in the revised manuscript, we believe this shows the default mode network plays a more important role in the network as a whole at the end of the dark period:

> Moreover, a more detailed examination of the top 15 hubs across time windows revealed a surprising pattern involving the default mode network (DMN) (Fig. 10a). In three of the four time windows—ZT0–4, ZT4–8, and ZT20–24—five of the top 15 hubs are DMN regions, whereas at ZT12–16, only two DMN regions appear among the top hubs (Fig. S11). Here we follow the anatomical definition of DMN based on the core regions outlined in [13]. Our findings here indicate that a more central position for the default mode network in the end of the dark period, right before the mice go to rest. This is confirmed by the calculation of the average centrality of all regions in the DMN (Fig. 10b), where a significantly higher average is shown at ZT20-24, compared to the value at ZT12-16. Different DMN regions' centrality is also shown in Fig. 10c.

Although we did not do the same for the salience network, which primarily consists of the anterior insular and the anterior cingulate cortex, we have found that the layer-specific active pattern of those two regions are quite different from other cortical regions, which we have pointed out in the result section:

> Although this trend was broadly observed across the cortex, there are some notable exceptions, including the anterior cingulate cortex (ACA) and the anterior insular cortex (AI) (Figs. S4 e-h). In particular, both regions(ACA and AI) are core components of the salience network [14], indicating a potential network phenomenon that we will analyze later.

We hope these have addressed your questions.

# 2 Reviewer 2

Point 2.1

The manuscript presents a modular framework for identifying active neuronal circuits under different temporal or behavioral conditions. The framework is a potentially valuable tool adaptable to various experimental setups such as, for example, IntelliCage (https://www.tse-systems.com/service/intellicage/, Kiryk et. al. 2020). The authors provide a proof-of-concept example of identifying neuronal networks predominantly active in daylight and nocturnal conditions.

Response 2.1

Thank you for your thoughtful summary and for highlighting the potential relevance of our work to other experimental pipelines, such as IntelliCage. In response to your suggestion, we have expanded the discussion section to include a consideration of how different experimental protocols and tools can be integrated with our framework.

> To include specific neuronal populations, alternative experimental designs involving labeling of active or inactive populations via staining of expressed proteins of interest can be of complementary value to the analysis conducted here. Moreover, although we chose to focus on four time windows around the 24-hour day with distinct circadian, light and sleep-wake driven behaviors, this framework can also be extended to study mouse brain connectivity under many other conditions, including neurodegenerative states, behavioral interventions, sleep deprivation, or even in combination with automated systems, such as the IntelliCage system [8].

Point 2.2

The building blocks of the proposed framework, such as labeling, imaging, and brain registration, are standard procedures. Although the workflow is well-arranged, it lacks a clear delineation of new possibilities that distinguish it from existing methodologies. The aspect of the study that I don't recall being presented or published anywhere else is integrating the CCF3 template with the excitatory/inhibitory neurons dataset and the presented TRAP2/CFOS activity data. Being a computational neuroscientist, I do understand the importance of data integration to fuel computational models with reliable data. The potential applicability is constrained by the availability of relevant datasets (Allen Institute mouse brain connectivity atlas and its derivatives, the atlas by Erö et al., 2018). The authors should better articulate the unique properties and potential outcomes offered by their proposed approach compared to alternative approaches to make it more appealing to a broader audience.

Response 2.2

Thank you for your comments. As you pointed out that "the potential applicability is constrained by the availability of relevant datasets," during our revision process, the spatial transcriptomic dataset [1] was made available. We quickly introduced a new method to

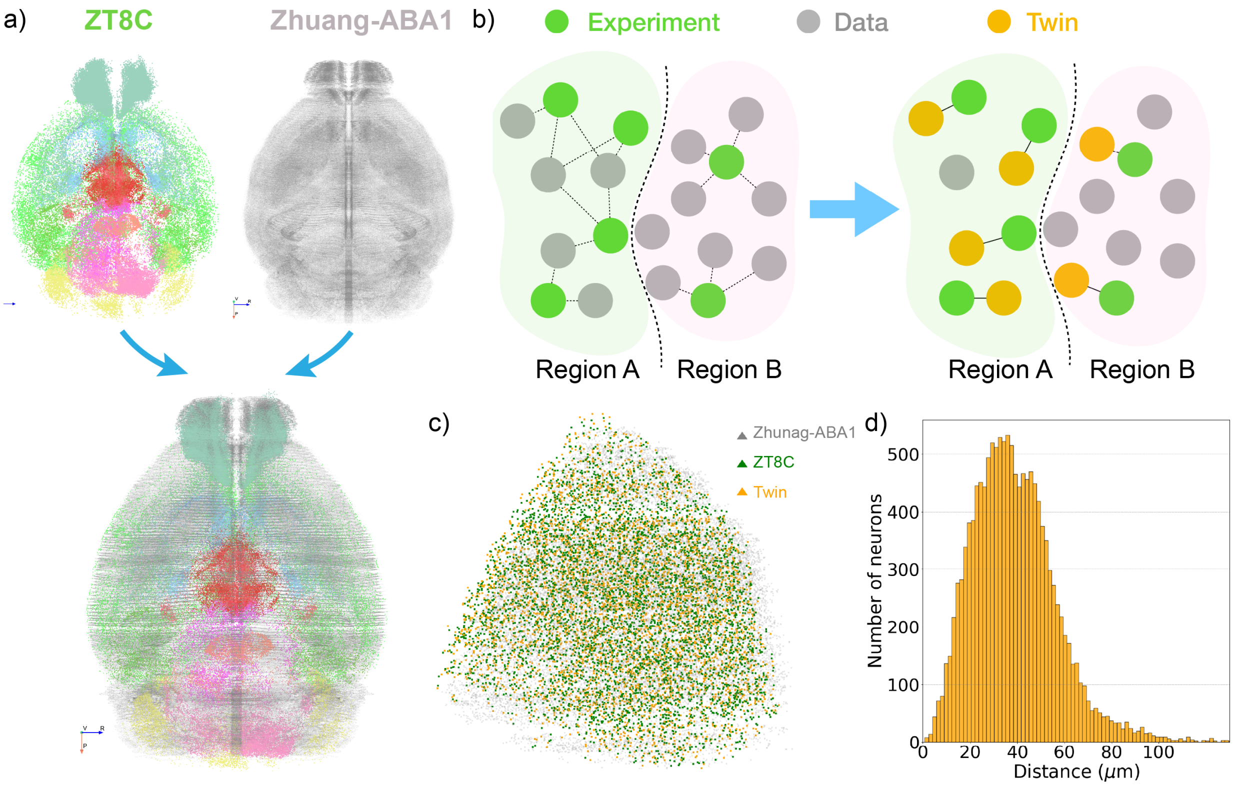


Figure 5: **Matching active neurons to the spatial transcriptomic dataset.** a) Spatial distribution of the neurons in Zhuang-ABA1(grey), our ZT8C brain(colored) and merged together in the same coordinate framework. b) Schematics of finding the twin neuron in the spatial transcriptomic dataset(Zhuang-ABA1) for each active neuron in our data based on spatial proximity. c) A demonstration of the neuron matching process with the right primary visual cortex of the ZT8C brain. Green points are our data, grey points are Zhuang-ABA1 and yellow points are the twin neurons after matching. d) The distribution of distances between the twin neurons in Zhunag-ABA1 and the neuron in our dataset for the ZT8C brain.

integrate this new dataset into our framework, which is illustrated in **Figure 5** attached above.

Compared to the original cell atlas [2], the new datasets have significantly increased the resolution of our previous procedures for identifying the neurotransmitter information of neurons identified in our dataset.

As more and more spatial transcriptomic datasets are becoming available, we believe this revision of our modular framework provides an opportunity to continuously integrate those new datasets, and the usability of our work will increase. As we wrote at the end of the manuscript:

> Multimodal data are available to the field of neuroscience at a surprising speed, creating new avenues for discoveries [3]. We believe that the experimental-

> computational pipeline presented in this study provides a strong example of how integrating multimodal data can advance the exploration and analysis of the mouse brain.

This is one of the unique contributions of our manuscript. Please refer to our response to the next point you raised for a more complete description of the innovations of our manuscript.

Point 2.3

Before addressing the minor details, I would recommend the Authors to: Clarify the novelty aspects of the manuscript, particularly regarding the proposed framework's unique contributions compared to existing methodologies.

Response 2.3

Thank you for raising this point. We have discussed the novelty aspects of the manuscript throughout the introduction and discussion sections. We would like to summarize these points here to answer your question. Methodologically, to our knowledge, our manuscript is the first to

1. Use c-fos expression to investigate the spontaneously active neurons on the whole-brain level at different times of the day.
2. Use spatial transcriptomic data to identify the molecular properties of neurons related to certain experimental protocols on the whole-brain level.
3. Introduce the definition of active connectivity, which integrates single-cell data with mesoscopic connectivity to make predictions.

These unique aspects of our framework help produce the following new discoveries, which we summarized at the beginning of the results section:

> Here, we introduced an experimental-computational pipeline to study the active neurons and networks in the mouse brain. As a proof of principle, we used the framework to investigate the active neurons and networks in the mouse brain at four different time windows of the day with distinct behaviors. We provided a whole-brain map of active neurons at those time windows, identified layer and region-specific variation of active neurons in the cortex, and observed an excitatory/inhibitory shift of active neurons in the whole-brain. Moreover, our construction of active connectivity has revealed the structural change of inter-regional communication between active neurons across the four time points of the day.

We hope these, along with our letter attached at the beginning of this response, have clarified our contributions.

Point 2.4

Consider providing critical data, algorithms, and results to enhance the reproducibility and usability of the study.

Response 2.4

We highly value the reproducibility and usability of our work. We have uploaded key data associated with this study, including the fluorescent images of all brains collected and the neuronal information used in the analysis, in the following repository: https://figshare.com/s/5c518ccaedc4dd5baeaa. We have also provided the details of computing and analyzing the active connectivity in the appendix. We hope you will find these resources helpful.

We will upload all the codes used in the work upon acceptance of the manuscript. We would also be willing to provide the code in advance upon your request.

# 3 Reviewer 3

Point 3.1

The goal of the study is to determine the difference in neuronal activity at night and during the day in the mouse brain. Active cells are tagged with by the cfos dependent Cre expression which enables tdT labeling in the presence of 4-hydroxytamoxifen. Labeled cells are visualized after tissue clearing of the whole brain and imaged with light sheet microscopy. The study finds that connection strength in insular cortex are stronger in the morning (light) while connections in the RSP and ACC are stronger in the evening (dark). The findings are compelling. The approach is innovative and has far-reaching potential for mapping functional connectivity of the mouse brain.

Response 3.1

Thank you for your accurate summary and for your kind remarks regarding our findings. We look forward to receiving your further feedback on the revised manuscript, as parts of the framework have been refined and new discoveries have emerged through these improvements.

Point 3.2

Although cfos expression has long been thought to be neuron specific, there is increasing evidence that it is also expressed in glial cells (astrocytes, oligodendrocytes, microglia). As glial cells play a role in sleep-wake regulation its seems reasonable to expect that some of the activated TRAP2-captured cells may not be neurons. I am aware that this is a tricky issue to resolve in the present study. Nevertheless the matter should be addressed and the potential implications should be considered in the discussion.

Response 3.2

Thank you for raising this important comment. Inspired by your comment, we have added a dedicated paragraph to discuss this. Please see:

> In the current study, we chose to utilize the well-known immediate early gene c-Fos as a driver to endogenously label active neurons. Although other cell types, such as glial cells, can also express c-Fos in the brain [15], previous validation experiments using the original fosTRAP1 system [16] demonstrated that more than 96% of TRAPed cells colocalized with NeuN, a neuron-specific marker. The more evolved fosTRAP2 system employed in our study is equally specific to neuronal populations, additionally preserving physiological c-Fos function via a 2A-linked iCre fusion [17]. We can thus safely assume that the large majority of captured cells in our study are neurons, and as previously shown, sufficient to recapitulate mammalian behaviors [18].

We hope this will address your concern.

Point 3.3

One of the interests that drives the study is to understand interactions between brain networks. To do this the authors introduce the term "active connectivity". Does this mean "functional connectivity"? Because the observations are based on static images of active cells, it is unclear how the data reveal time-sensitive correlations between seeds and the interacting targets.

Response 3.3

Thank you for raising this important question, which is central to our manuscript.

First, we have refined our definition of active connectivity in the revised manuscript. The central questions we think are: knowing the number of active neurons in each region and the underlying mesoscopic structural connectivity, how can we measure the structural change of the networks formed by these active neurons? How do the roles of different regions shift in this network at different times of the day? To answer these questions, we refined our definition of active connectivity. Compared to the original manuscript, we no longer build a neuron-to-neuron connectome between active neurons, but build a mesoscopic, regional active connectivity matrix for each brain. This improvement makes it possible to use established tools from network science to systematically analyze the derived active connectivity across different time windows. Please also see our response to your next point for a more detailed description of how the new active connectivity is constructed.

Second, to our understanding, active connectivity differs from functional connectivity in several key aspects, and also shares certain similarities. We have added a dedicated paragraph in the Discussion section to address this comparison in detail. Please see:

> Since functional connectivity is a widely used measure for studying interactions between brain regions, here we compare active connectivity with functional connectivity, which are both related to structural connectivity. Compared to the

unclear, nonlinear relationship between functional and structural connectivity [19, 20], the relationship between active connectivity and structural connectivity is much more straightforward mathematically. Functional connectivity is typically calculated as the correlation between regional activity time series recorded with techniques such as functional MRI (fMRI), resulting in a symmetric (undirected) correlation matrix. In contrast, active connectivity, as defined here, is inherently directed, following the source-target relationships present in the structural connectivity. Furthermore, functional connectivity captures dynamic interactions over short time windows—on the order of seconds to minutes—whereas active connectivity, defined in this work, reflects an integration of neuronal activity over a much longer time frame (approximately four hours in our experiments). Thus, while functional connectivity excels at detecting fast, transient interactions, active connectivity assesses more sustained patterns of network engagement, more causally anchored to the structural connectivity. From the perspective of neuronal plasticity, mechanisms such as spike-timing-dependent plasticity (STDP) require coincident high-level activity in both pre- and postsynaptic neurons [21]. Since our approach captures the most active neurons in the mouse brain at a given time, the active connectivity defined here may also highlight connections that are most likely undergoing synaptic changes, offering a complementary view of the brain's adaptive network dynamics.

We hope these have clarified your question.

Point 3.4

More specifically, It requires explanation how "connectivity" can be inferred from the present data. Unless I am missing a key point of the analysis, I am skeptical that connectivity can be established from the spatial distribution of differentially active cells. The study by Knox et al., (2018), which is foundational to the approach used in this paper, used data of anterograde viral tracing to map the mouse mesoscale connectome. Even If I am the only one who is confused here, a more systematic explanation of the magical machine-learning approach for extracting connection probability from the location of voxels aligned to the ABA-CCF atlas may be helpful.

Response 3.4

Thank you for raising this question. We apologize that we did not clearly explain how the active connectivity was constructed in the original manuscript. First, the machine learning algorithm was only used in segmenting the active neurons from the fluorescent images, but not in constructing the active connectivity. Secondly, as we mentioned previously, we no longer build a neuron-to-neuron connectome between active neurons, but build a mesoscopic, regional active connectivity matrix for each brain.

In the revised manuscript, **Figure 7** (attached below) and the result section have been rewritten to fully explain the implication of the active connectivity and how it is con-

structed. To summarize, we came up with a metric that measures the communication between active neurons in regions A and B, based on the following three quantities:

1. Regions A and B are structurally connected with a connection density $\rho_{AB}$,
2. Region A contains a sufficient number of active neurons ($N_A$) to send signals,
3. Region B contains active neurons ($N_B$) receiving the signal and sending it to a different region.

We then assume the "active connection strength" between region A and region B is the product of these quantities: $W_{AB} = N_A \times \rho_{AB} \times N_B$. This definition will result in a different connectivity matrix for each brain, which can be analyzed like other studies have done for the structural connectivity. [5] [6] We have done so in this manuscript and have revealed many insights about how the network formed by active neurons changes across different times of the day. Please see the appendix for more details about the construction and analysis of active connectivity.

We hope these have addressed your questions.

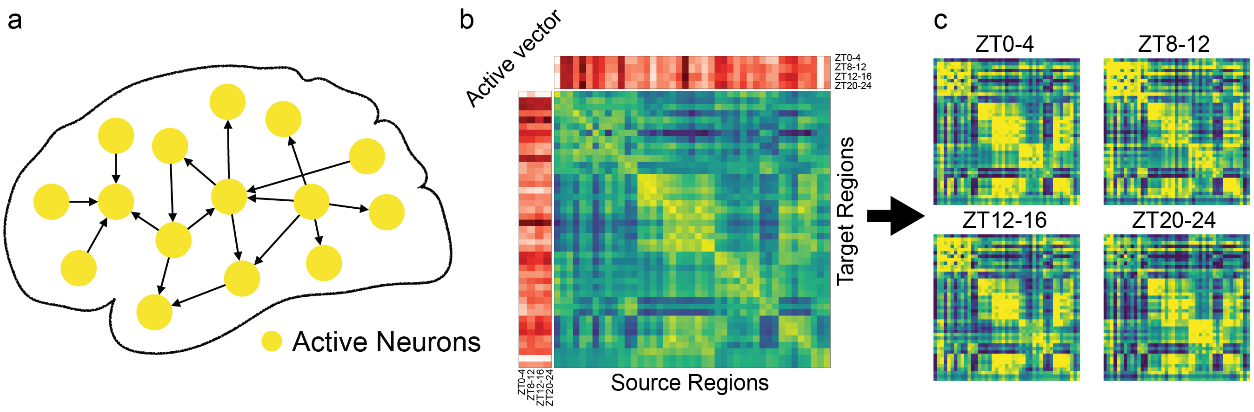


Figure 7: **Construction of the active connectivity.** The active connectivity is measuring the strength of communication between active neurons in different regions (a). By using the normalized connection density between different regions and the regional information of active neurons (b), we attain different active connectivity matrices for different time windows (c).

Point 3.5

It is stated as a matter of fact that during the day mice are less active and sleep more than at night (page 10). As this is a key argument for interpreting the data, it seems necessary to demonstrate whether this is indeed the case.

Response 3.5

Thank you for raising this question. The original statement might be too general and lacks statistical support since we only had two animals for each time point. This finding has been improved in the revised manuscript and is shown in **Figure 2** (attached below).

Since we have increased the number of time points from two to four, we are now very specific about which time points are being compared. We have also increased samples at each time window to offer stronger statistical support for our findings. The revised result section is attached here:

> We see a variation in the total number of neurons that are identified, with most occuring at ZT20-24 and ZT 0-4 (Fig. 2b). To avoid potential discrepancies between different images based on the experimental procedures, we calculated the ratio of the number of neurons in each region to the total number of neurons in a brain to investigate its regional composition (Fig. 2c and Fig. S2). Among the different major brain regions, we found that both the proportion and absolute number Fig. S2 of active neurons in the isocortex are significantly higher at ZT0-4, a period with a high amount of sleep compared to ZT12-16, compared to a period when the animals are waking from the rest period (Fig. 2c). To our surprise, the isocortex is the only major subregion that shows significant change in the number of active neurons.

In summary, the brain as a whole exhibit some differences, though not significant, in the number of active neurons across the four time points. However, the cortex shows a clear and significant variation, both in the absolute number of active neurons and in the proportion of cortical neurons relative to the total population. We hope this clarifies your question.

### Point 3.6

In the Discussion I would recommend to either separate references to the human sleep literature or make it absolutely clear to what species the text referrers to.

### Response 3.6

Thank you for your comment, and we agree that the discussion should be clear on which species are discussed. We have carefully rewritten the discussion section, and it almost contains only references to the mouse literature. We hope this has been clarified.

### Point 3.7

Figure 2j shows a bar plot of active neurons at light onset and light offset in different brain areas. It would be helpful to show these areas in the Figure 2i. In addition, the areas should be described in a list of abbreviations. Figure 2j might be improved by showing all areas considered at ZT0 and ZT12 on the X axis and plot the activity ratios at each time point side-by-side.

### Response 3.7

Thank you for your suggestion on Figure 2. Since we now have four time points, the original way of producing Figure 2i does not work anymore. Instead, we plot the most active regions at each time window based on the regional over the whole brain or its mother structure. Figure 2j was replaced by a series of plots, such as Figure 3a and Figure S4,

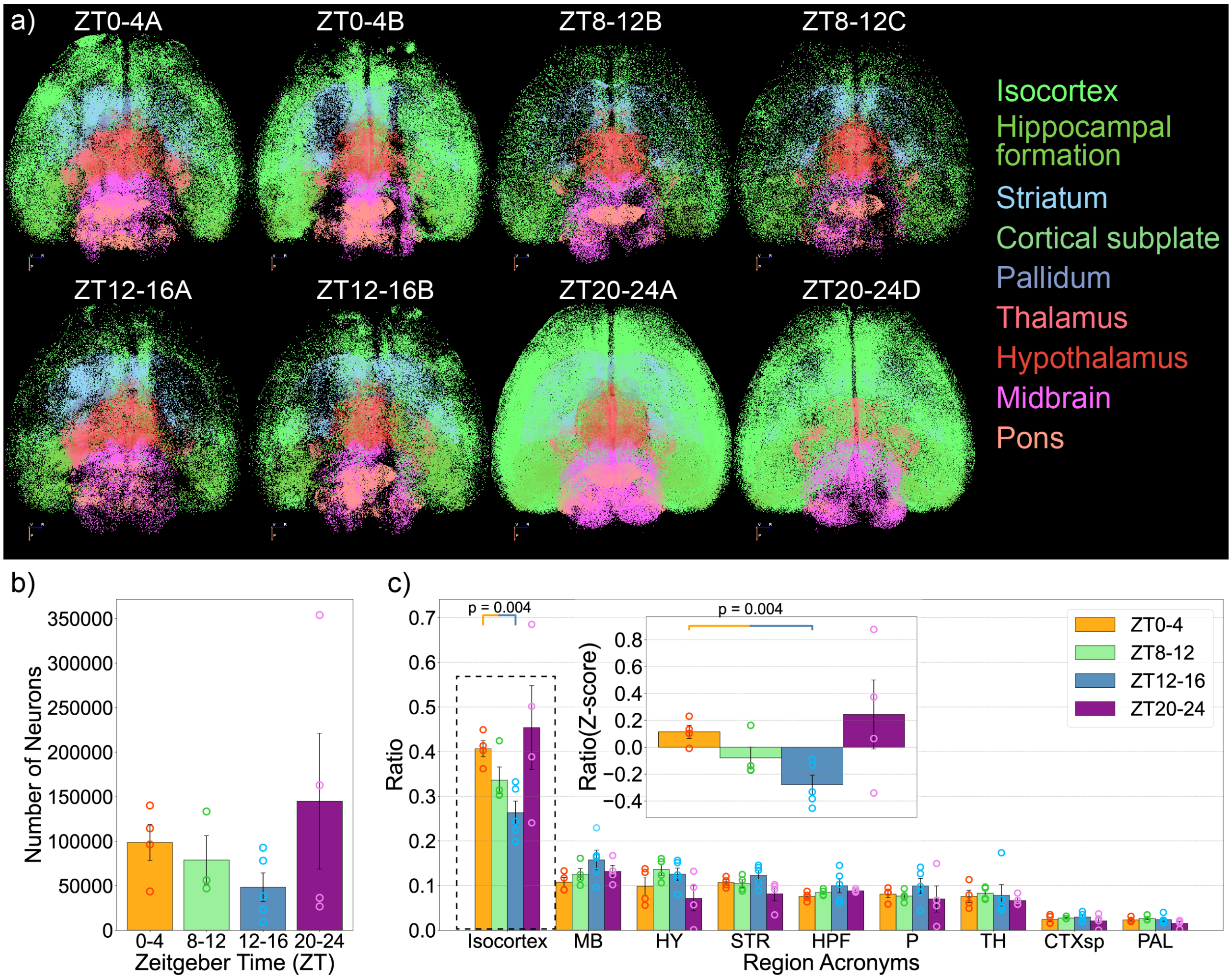


Figure 2: **Variation of active neurons in the whole brain across different time windows** a) Visualizations of neurons in major brain regions for each time window(2 brains for each time point). The full visualizations of all brains and all brain regions(including the cerebellum, olfactory areas, and the medulla) are presented in Fig. S2. b) The total number of active neurons in the brain at different time windows during the day. c) Ratios of different major brain regions for different time windows(ratio is calculated by dividing the number of neurons in each region by the total number of neurons). The ratio of the isocortex is significantly higher at ZT0-4 compared to ZT12-16(p-value = 0.004), which is further z-scored in the panel inside. 6

where the most active regions in the cortex and other different major brain regions are shown.

Point 3.8

In Figure 3a, 3b what is AII? I assume it stands for ”all”. In order to avoid confusion revise the font.

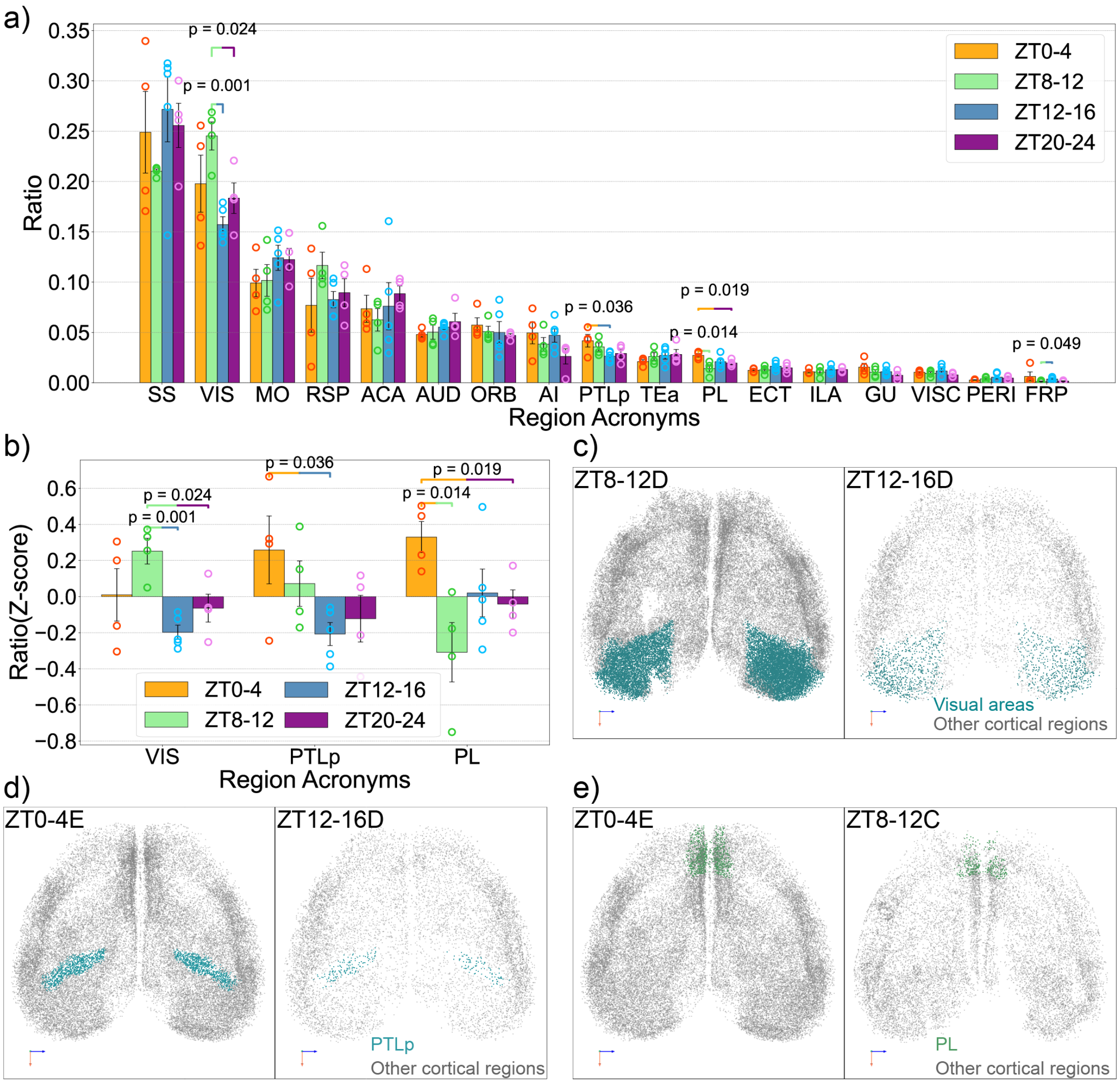


Figure 3: **Region-specific variation of active neuron distribution in the cortex.** a) Ratios of major cortical regions over the whole cortical population. b) The three regions that show significant differences in their ratios over the total cortical population across different times (VIS: Visual areas, PTLp: Posterior parietal association area, PL: Prelimbic areas). Here, the ratios are Z-scored based on the average of each region to show the differences between different times. c) Visualizations of active neurons in the visual regions for ZT8-12D and ZT12-16D brain. d) Visualizations of active neurons in the visual regions for ZT0-4E and ZT12-16D brain. e) Visualizations of active neurons in the visual regions for ZT0-4E and ZT8-12C brain.

Response 3.8

We apologize for the confusion caused by the legend. In the original Figure 3, "All" means all the other neurons. The revised Figures 3c-e have similar plots of active neurons, and we have changed the legend to "other cortical regions".

Point 3.9

Figure 3e Revise legend to "project to and receives input from". The readily accessible information content would be improved by plotting the connectivity strengths for each time point and area side-by-side.

Response 3.9

Thank you again for your suggestion on the legend of the figure. However, we have omitted Figure 3e in the revised manuscript.

Point 3.10

P2 L4 Please explain what "how they interact" means.

Response 3.10

We apologize for the vagueness in our original expression. The abstract has been carefully rewritten, and this phrase has been omitted. In the original expression, we were referring to the synaptic activities between the active neurons. Please see our response in the next point for the revised abstract.

Point 3.11

P2 L9 The sentence is awkward. Please revise to indicate more clearly that the interest was to determine which networks were active at night and during the day and on which parameters the strength of the networks depend.

Response 3.11

Thank you for pointing this out. The original sentence has been removed in the revised manuscript. Please see the attached for our new abstract:

> The mouse brain's activity changes drastically over a day despite being generated from the same neurons and physical connectivity. To better understand this, we develop an experimental-computational pipeline to determine which neurons and networks are most active at different times of the day. We genetically mark active neurons of freely behaving mice at four times of the day with a c-Fos activity-dependent TRAP2 system. Neurons are imaged and digitized in 3D, and their molecular properties are inferred from the latest brain spatial transcriptomic dataset. We then develop a new computational method to analyze the network formed by the identified active neurons. Applying this pipeline, we observe region and layer-specific activation of neurons in the cortex, especially activation of layer five neurons at the end of the dark (wake) period. We also observe a shift in the balance of excitatory (glutamatergic)

> neurons versus inhibitory (GABAergic) neurons across the whole brain, especially in the thalamus. Moreover, as the dark (wake) period progresses, the network formed by the active neurons becomes less modular, and the hubs switch from subcortical regions, such as the posterior hypothalamic nucleus, to cortical regions in the default mode network. Taken together, we present a pipeline to understand which neurons and networks may be most activated in the mouse brain during an experimental protocol, and use this pipeline to understand how brain activity changes over the course of a day.

**Point 3.12**

12 P2 L23 I have trouble understanding how snapshots of the active cells can reveal how the cells interact.

**Response 3.12**

Thank you for your question. The original statement is now deleted from the main text, but we would like to briefly explain since this is related to the idea of the active connectivity. As we wrote in the manuscript:

> To conceptualize communication from one brain region to another, consider the scenario where signals are sent from region A to region B, which has a structural connection density denoted as $\rho_{AB}$. If $\rho_{AB}$ is very low, indicating a lack of structural connectivity, then no signal transmission is expected. However, even when substantial structural connections exist, if region A lacks active neurons, no signal can be initiated. Similarly, if region B lacks active neurons, incoming signals may not be propagated further effectively. Based on this reasoning, we define the "active" connection strength from active neurons in region A to those in region B as the product of three factors: the average structural connection density $\rho_{AB}$, the number of active neurons in region A, and the number of active neurons in region B (Fig. 7a).

We agree with your statement that it is very difficult to infer how individual neurons interact with each other with the information provided by our experiments. Therefore, our refined definition of active connectivity is based on a regional resolution, measuring an average of possible communication between active neurons in different regions at different times of the day.

**Point 3.13**

P3 L1 Please provide a more explicit justification for the study than "understudied". List the open questions and why they are worth to be answered.

**Response 3.13**

Thank you for pointing this out. The original sentence has been removed in the revised manuscript and the introduction has been carefully rewritten to avoid such vagueness.

# References


[1] Zhang M, Pan X, Jung W, Halpern AR, Eichhorn SW, Lei Z, et al. Molecularly defined and spatially resolved cell atlas of the whole mouse brain. Nature. 2023 Dec;624(7991):343-54. Available from: https://www.nature.com/articles/s41586-023-06808-9.

[2] Erö C, Gewaltig MO, Keller D, Markram H. A Cell Atlas for the Mouse Brain. Frontiers in Neuroinformatics. 2018;12. Available from: https://www.frontiersin.org/article/10.3389/fninf.2018.00084.

[3] Arkhipov A, da Costa N, de Vries S, Bakken T, Bennett C, Bernard A, et al. Integrating multimodal data to understand cortical circuit architecture and function. Nature Neuroscience. 2025;28(4):717-30. Available from: https://doi.org/10.1038/s41593-025-01904-7.

[4] Mitani TT, Susaki EA, Matsumoto K, Ueda HR. Realization of cellomics to dive into the whole-body or whole-organ cell cloud [Journal Article]. Nature Methods. 2024;21(7):1138-42. Available from: https://doi.org/10.1038/s41592-024-02307-5.

[5] Fulcher BD, Fornito A. A transcriptional signature of hub connectivity in the mouse connectome [Journal Article]. Proc Natl Acad Sci U S A. 2016;113(5):1435-40. 1091-6490 Fulcher, Ben D Fornito, Alex Journal Article Research Support, Non-U.S. Gov't United States 2016/01/17 Proc Natl Acad Sci U S A. 2016 Feb 2;113(5):1435-40. doi: 10.1073/pnas.1513302113. Epub 2016 Jan 15.

[6] Coletta L, Pagani M, Whitesell JD, Harris JA, Bernhardt B, Gozzi A. Network structure of the mouse brain connectome with voxel resolution. Science Advances. 2020;6(51). Available from: <GotoISI>://WOS:000599905500008.

[7] Herculano-Houzel S, Mota B, Lent R. Cellular scaling rules for rodent brains [Journal Article]. Proceedings of the National Academy of Sciences. 2006;103(32):12138-43. Available from: https://www.pnas.org/doi/abs/10.1073/pnas.0604911103.

[8] Kiryk A, Janusz A, Zglinicki B, Turkes E, Knapska E, Konopka W, et al. IntelliCage as a tool for measuring mouse behavior – 20 years perspective. Behavioural Brain Research. 2020;388:112620. Available from: https://www.sciencedirect.com/science/article/pii/S0166432820303193.

[9] Kosmidis S, Negrean A, Dranovsky A, Losonczy A, Kandel ER. A fast, aqueous, reversible three-day tissue clearing method for adult and embryonic mouse brain and whole body. Cell Reports Methods. 2021;1(7):100090. Available from: https://www.sciencedirect.com/science/article/pii/S2667237521001454.

[10] Williams B, Del Rosario J, Muzzu T, Peelman K, Coletta S, Bichler EK, et al. Spatial modulation of dark versus bright stimulus responses in the mouse visual system. Curr Biol. 2021;31(18):4172-9.e6.

[11] Harris JA, Mihalas S, Hirokawa KE, Whitesell JD, Choi H, Bernard A, et al. Hierarchical organization of cortical and thalamic connectivity. Nature. 2019;575(7781):195+. Available from: `<GotoISI>://WOS:000496159900061https://www.nature.com/articles/s41586-019-1716-z.pdf`.

[12] Oh SW, Harris JA, Ng L, Winslow B, Cain N, Mihalas S, et al. A mesoscale connectome of the mouse brain. Nature. 2014;508(7495):207-14. Available from: `https://dx.doi.org/10.1038/nature13186https://www.nature.com/articles/nature13186.pdf`.

[13] Whitesell JD, Liska A, Coletta L, Hirokawa KE, Bohn P, Williford A, et al. Regional, Layer, and Cell-Type-Specific Connectivity of the Mouse Default Mode Network. Neuron. 2021;109(3):545-59.e8. Available from: `https://www.sciencedirect.com/science/article/pii/S0896627320308898`.

[14] Mandino F, Vrooman RM, Foo HE, Yeow LY, Bolton TAW, Salvan P, et al. A triple-network organization for the mouse brain. Molecular Psychiatry. 2022;27(2):865-72.

[15] Cruz-Mendoza F, Jauregui-Huerta F, Aguilar-Delgadillo A, García-Estrada J, Luquin S. Immediate Early Gene c-fos in the Brain: Focus on Glial Cells [Journal Article]. Brain Sci. 2022;12(6). 2076-3425 Cruz-Mendoza, Fernando Jauregui-Huerta, Fernando Orcid: 0000-0002-3902-9009 Aguilar-Delgadillo, Adriana García-Estrada, Joaquín Luquin, Sonia Journal Article Review Switzerland 2022/06/25 Brain Sci. 2022 May 24;12(6):687. doi: 10.3390/brainsci12060687.

[16] Guenthner CJ, Miyamichi K, Yang HH, Heller HC, Luo L. Permanent genetic access to transiently active neurons via TRAP: targeted recombination in active populations. Neuron. 2013;78(5):773-84.

[17] Onishi T, Hirose K, Sakaba T. Molecular tools to capture active neural circuits [Journal Article]. Front Neural Circuits. 2024;18:1449459. 1662-5110 Onishi, Taichi Hirose, Kenzo Sakaba, Takeshi Journal Article Review Switzerland 2024/08/05 Front Neural Circuits. 2024 Jul 19;18:1449459. doi: 10.3389/fncir.2024.1449459. eCollection 2024.

[18] DeNardo LA, Liu CD, Allen WE, Adams EL, Friedmann D, Fu L, et al. Temporal evolution of cortical ensembles promoting remote memory retrieval. Nature Neuroscience. 2019;22(3):460-9.

[19] Deco G, Ponce-Alvarez A, Mantini D, Romani GL, Hagmann P, Corbetta M. Resting-state functional connectivity emerges from structurally and dynamically shaped slow linear fluctuations [Journal Article]. J Neurosci. 2013;33(27):11239-52.

[20] Zhou C, Zemanová L, Zamora G, Hilgetag CC, Kurths J. Hierarchical Organization Unveiled by Functional Connectivity in Complex Brain Networks [Journal Article]. Physical Review Letters. 2006;97(23):238103. PRL. Available from: `https://link.aps.org/doi/10.1103/PhysRevLett.97.238103`.

[21] Feldman DE. The spike-timing dependence of plasticity [Journal Article]. Neuron. 2012;75(4):556-71. 1097-4199 Feldman, Daniel E R01 NS073912/NS/NINDS

NIH HHS/United States R01 073912/PHS HHS/United States Journal Article Research Support, N.I.H., Extramural Research Support, U.S. Gov't, Non-P.H.S. Review United States 2012/08/28 Neuron. 2012 Aug 23;75(4):556-71. doi: 10.1016/j.neuron.2012.08.001.
