# Supplementary material for: A framework to determine active neurons and networks within the mouse brain reveals how brain activity changes over the course of the day
Source: PLoS Biol. 2025 Nov 13;23(11):e3003472. doi: 10.1371/journal.pbio.3003472 (PMC12633928; doi:10.1371/journal.pbio.3003472)
Supplement: Attachment — Submitted filename: Response to Reviewers.pdf [file pbio.3003472.s003.pdf]

Response to Second Round of Reviews for Manuscript: A framework to determine active neurons and networks within the mouse brain

Guanhua Sun[1], Tomoyuki Mano[4, 1], Shoi Shi[4], Alvin Li[1], Koji L Ode[4], Alex Rosi-Andersen[3], Erica Pedron[3], Steven A Brown[†3], Hiroki R Ueda[4, 5, 6], Konstantinos Kompotis[*3, 1], and Daniel Forger[*1, 2]

[1]Department of Mathematics, University of Michigan, Ann Arbor, United States
[2]Gilbert S. Omenn Department of Computational Medicine and Bioinformatics, University of Michigan, Ann Arbor, United States
[3]Chronobiology and Sleep Research Group, Institute of Pharmacology and Toxicology, University of Zurich, Zurich, Switzerland
[4]Department of Systems Pharmacology, Graduate School of Medicine, the University of Tokyo, Tokyo, Japan
[5]Laboratory of Synthetic Biology, RIKEN Biosystems Dynamics Research, Osaka, Japan
[6]Department of Systems Biology, Institute of Life Science, Kurume University, Fukuoka, Japan

† In memoriam
∗ To whom correspondence should be addressed

Letter to Reviewers and Editors

Dear Reviewers and Editors at PLOS Biology:

Thank you for reviewing the revision of our manuscript! We sincerely appreciate the time and effort you have dedicated to this process.

We are pleased to hear that the major concerns raised in the previous round have been satisfactorily addressed. In this revision, we have made additional changes to respond to the remaining reviewer comments. We have also carefully edited the manuscript for improved clarity and language throughout.

To further enhance reproducibility, we have uploaded a more complete set of materials, including the full codes used to generate the results presented in the manuscript, to the following repository: https://figshare.com/s/5c518ccaedc4dd5baeaa.

Please find below our point-by-point responses to the remaining comments:

Point-to-point response to reviews

1 Reviewer 2

The Authors have significantly remodeled the manuscript, increased the number of experiments and time windows, and refined the methods. Most importantly, however, the authors fully addressed the issues regarding the novelty elements of the study by including a mouse spatial transcriptomic dataset, among other actions. I have also inspected the provided TIFF stacks and CSV tables, and they meet the minimum required standards. I have no further remarks regarding the manuscript.

Response 1.1

We thank you for your careful reading of our revised manuscript. We are grateful for your recognition of our use of the spatial transcriptomic dataset, which we believe represents a novel and impactful approach that will contribute to future research in this field.

To enhance reproducibility, we have uploaded all relevant materials, including code and data, to the following public repository: https://figshare.com/s/5c518ccaedc4dd5baeaa.

2 Reviewer 4

In the study the authors "introduced an experimental-computational pipeline to study the active neurons and networks in the mouse brain". I believe this is a very nice and important tool, and the application used is very interesting.

I have to emphasize that I find it difficult as a reviewer to come into the 2nd round of reviews, as asking for extensive new analyses is not ideal at this stage. The authors have done a great job answering the prior reviewer question. However, I believe some important limitations

remain, and are not clearly mentioned in the paper. I would like to ask the authors for a few small edits to point them out clearly and help clarify the manuscript.

Point 2.1

The method presented, while great for homogeneous areas, will likely mix cells and produce statistical averages across transcriptomically heterogeneous areas. In Section 2.4: the areas analyzed are relatively homogeneous transcriptomically. The authors should either include analysis of areas with heterogeneous cell types (e.g. most cortical L2/3 areas), or clearly state this as a limitation clearly at the end of 2.4. This limitation is briefly mentioned later in the discussion, but I think it should be present when presenting the results. As a result of limitation 1, the conclusion that in the cortex there is relative stability of E/I activation can be simply a lack of capacity of the method to tell apart e/i cells in cortex. I did not see a mention to this limitation.

Response 2.1

Thank you for raising this important point. We agree with the reviewer that our method has practical limitations when applied to regions with highly heterogeneous transcriptomic profiles, such as cortical layers 2/3, as the reviewer has pointed out. We acknowledged this limitation in the Discussion section of the previous revision and agree that it is worths further emphasis. In response to the reviewer's suggestion, we have now highlighted this limitation more explicitly in the main text. Please see the following for the revised paragraph in the Results section:

> This regional shift may reflect an increased demand for sleep-related processes late in the day, as inhibitory populations such as those in the reticular nucleus become more active. This result may also be influenced by the relatively uniform neuronal composition observed in regions such as the sensory thalamic nuclei. In contrast, areas like cortical layers 2/3 exhibit a more heterogeneous and intermixed distribution of neuronal subtypes, which may reduce the accuracy of the method.

We also further elaborated on this limitation in the revised Discussion section. In particular, we emphasize that the challenges associated with heterogeneous transcriptomic regions may be alleviated in the future through the use of emerging spatial transcriptomic technologies with higher cellular resolution. We have also pointed out the observation that cortical E/I balance stays relatively stable might be caused by a methodological discrepancy between different approaches of identifying E/I balance. Please see the following excerpt from the updated Discussion section:

> The regional analysis of active neurons was followed by alignment to the spatial transcriptomic data by finding the closest neighbor in the dataset (Zhuang-ABA1). Although the spatial distribution between neurons in our dataset and the matched neurons is relatively close, the method can still produce discrepancies, in particular, for those regions where different types of neurons are tightly mixed, such as the layer 2/3 of the cortex. In our cell-type-specific analysis of the cortex, we observed that the excitatory-to-inhibitory (E/I) ratio of ac-

> tive neurons remains relatively constant. This finding stands in contrast to several previous studies that report dynamic changes in E/I balance in the cortex [1, 2, 3]. This discrepancy can be explained by the error in our method when applied to heterogeneous regions, but can also arise from methodological differences: whereas prior studies typically infer E/I balance from firing activity based on electrophysiological recording, our analysis directly quantifies the number of excitatory and inhibitory neurons. On the other hand, our method will work especially well for brain regions where different types of neurons are more spatially segregated, such as the thalamus or hypothalamus. In the future, we would expect more spatial transcriptomic data with higher resolution that can be seamlessly used in the framework presented in this work to further increase the accuracy of identifying cell properties [4].

Point 2.2

The authors mention: "Although a more recent update to this dataset is available [3], providing layer-specific and cell-type-specific projection information, it is limited to the thalamocortical network and therefore was not used for the whole-brain analyses conducted in this study." There is a newer connectivity model (Koelle et al. 2023) which is cell-type specific and extends the work from Harris et al 2019 (the reference 3) to the whole-brain. I presume the authors constructed their framework before the 2023 model was available. If this is the case, please mention it. If not, do please explain why the older model (Knox et al. 2018) was used.

Response 2.2

We thank the reviewer for bringing up this important point. As noted, our framework was developed and the original manuscript submitted prior to the release of the 2023 model. The introduction of this updated model presents a valuable opportunity to further strengthen our study and also highlights the extensibility of our framework to incorporate emerging datasets. We have now included a citation to this new resource and have revised the Discussion section accordingly to reflect its relevance.

Please see below for the revised text in the corresponding section:

> To construct the active connectivity, we utilized one of the early versions of the whole-brain mesoscopic connectivity dataset [5]. Although a more recent update to this dataset is available [6], providing layer-specific and cell-type-specific projection information, it is limited to the thalamocortical network and therefore was not used for the whole-brain analyses conducted in this study [7, 8]. We also did not incorporate the voxelized extension of the connectivity data, which could significantly improve the spatial resolution of the results presented in this work. Integrating these resources represents an important direction for future work.

Point 2.3

There isn't a clear limitations section or paragraph in the discussion, which would help the clarity. Minor; reference compilation error on Line 412. The changes mentioned are small enough to not require the paper to be resent to the reviewer.

Response 2.3

We thank the reviewer for raising these thoughtful points. We have completed the suggested minor corrections. While we did not further subdivide the Discussion section into multiple subsections, we have expanded the discussion of the limitations of our method, which is related to the responses to the previous points.

3 Academic Editor

Point 3.1

I appreciate that in the Discussion the authors try to contrast what they call active connectivity (which by their definition is a necessary condition for inter-regional communication) with traditionally established functional connectivity, as defined by time series correlations. Given that in the revised work, they consider activity for more time points, they may perhaps also be able to derive a correlation based functional connectivity measure for the data, in order to compare it directly with active connectivity. However, if it is not practical, this direct comparison could also be left for future work and just be mentioned as an outlook.

Response 3.1

We sincerely appreciate the insightful comment from the Academic Editor. During the revision process, we explored the possibility of computing functional connectivity from our dataset. We did some preliminary analyses, but eventually decided to leave this for future work.

That said, we fully agree that comparing the functional connectivity with the active connectivity defined in this work at different time windows would be highly meaningful. In response to this suggestion, we have expanded the Discussion section to acknowledge this point and highlight it as an important direction for future research.

Please see below for the revised text in the corresponding section of the manuscript:

> Furthermore, functional connectivity captures dynamic interactions over short time windows, on the order of seconds to minutes, whereas active connectivity, defined in this work, reflects an integration of neuronal activity over a much longer time frame (approximately four hours in our experiments). Thus, while functional connectivity excels at detecting fast, transient interactions, active connectivity assesses more sustained patterns of network engagement, more causally anchored to the structural connectivity. Nevertheless, it would be valuable for future studies to perform fMRI studies across different circa-

dian time windows and to quantify the corresponding functional connectivity. Comparing these with the active connectivity defined in the present study may yield important insights into the temporal organization of brain networks.

References

[1] Kushinsky D, Tsivourakis E, Apelblat D, Roethler O, Breger-Mikulincer M, Malina KCK, et al. Daily light-induced transcription in visual cortex neurons drives downward firing rate homeostasis and stabilizes sensory processing. Cell Reports. 2024;43(9).

[2] Zong FJ, Min X, Zhang Y, Li YK, Zhang XT, Liu Y, et al. Circadian time-and sleep-dependent modulation of cortical parvalbumin-positive inhibitory neurons. The EMBO Journal. 2023;42(3):e111304.

[3] Bridi MCD, Zong FJ, Min X, Luo N, Tran T, Qiu J, et al. Daily Oscillation of the Excitation-Inhibition Balance in Visual Cortical Circuits. Neuron. 2020;105(4):621-9.e4.

[4] Mitani TT, Susaki EA, Matsumoto K, Ueda HR. Realization of cellomics to dive into the whole-body or whole-organ cell cloud [Journal Article]. Nature Methods. 2024;21(7):1138-42. Available from: https://doi.org/10.1038/s41592-024-02307-5.

[5] Oh SW, Harris JA, Ng L, Winslow B, Cain N, Mihalas S, et al. A mesoscale connectome of the mouse brain. Nature. 2014;508(7495):207-14. Available from: https://dx.doi.org/10.1038/nature13186https://www.nature.com/articles/nature13186.pdf.

[6] Harris JA, Mihalas S, Hirokawa KE, Whitesell JD, Choi H, Bernard A, et al. Hierarchical organization of cortical and thalamic connectivity. Nature. 2019;575(7781):195+. Available from: <GotoISI>://WOS:000496159900061https://www.nature.com/articles/s41586-019-1716-z.pdf.

[7] Knox JE, Harris KD, Graddis N, Whitesell JD, Zeng HK, Harris JA, et al. High-resolution data-driven model of the mouse connectome. Network Neuroscience. 2018;3(1):217-36. Available from: <GotoISI>://WOS:000472684700003https://www.ncbi.nlm.nih.gov/pmc/articles/PMC6372022/pdf/netn-03-217.pdf.

[8] Koelle S, Mastrovito D, Whitesell JD, Hirokawa KE, Zeng H, Meila M, et al. Modeling the cell-type-specific mesoscale murine connectome with anterograde tracing experiments. Network Neuroscience. 2023;7(4):1497-512.
